# Error in Box 1

**DOI:** 10.1001/jamanetworkopen.2023.32858

**Published:** 2023-08-24

In the Consensus Statement titled “Guidelines for Diagnosis and Management of Infective Endocarditis in Adults: A WikiGuidelines Group Consensus Statement,”^1^ published July 31, 2023, there was an error in Box 1. In the description of vancomycin as a principal agent for native valve infective endorcarditis, *Staphylococcus aureus* originally appeared as *Streptococcus aureus.* This article has been corrected.^1^
